# Effects of Posttraumatic Stress Disorder and Mental Disorders on the Labor Market Integration of Young Syrian Refugees

**DOI:** 10.3390/ijerph20032468

**Published:** 2023-01-30

**Authors:** Hans Dietrich, José Luis Álvaro Estramiana, Alicia Garrido Luque, Volker Reissner

**Affiliations:** 1 Institute for Employment Research (IAB), Regensburger Str. 104, 90478 Nürnberg, Germany; 2 Departamento de Antropología Social y Psicología Social, Universidad Complutense de Madrid, 28223 Madrid, Spain; 3 Department of Child and Adolescent Psychiatry and Psychotherapy, University of Duisburg-Essen, Wickenburgstr. 21, 45147 Essen, Germany

**Keywords:** PTSD-ETI, anxiety and depression HSCL-10, Syrian refugees, labor market integration

## Abstract

Civil war experience in the Syrian home country, insecurity and critical life events during migration, or adverse events in the receiving country might affect refugees’ mental health. This paper addresses the effects of psychological distress and mental disorders on refugees’ labor market integration in Germany between 2016 and 2021. We employ survey data from about 2700 young Syrians, delivering information on individuals’ experience of migration and arrival in Germany in 2016. The survey data were successfully merged with register data, delivering detailed information regarding individuals’ process of labor market integration and employment status from 2016 to 2021. Overall, the labor market integration of young refugees improved remarkably over time. In 2021, about 69% of the study population was integrated in a wider sense, and 30% was employed in fulltime contracts in 2021. However, the results indicate long-lasting effects of PTSD and mental disorders on individuals’ labor market integration, whilst individuals’ characteristics related to migration and arrival lose relevance over time and hardly affect labor market integration around five years after arrival. High PTSD scores in 2016 indicate a significantly reduced full-time employment probability in 2021. Anxiety and depression show significant negative effects on individuals’ labor market integration, but with a less severe impact compared to a PTSD diagnosis.

## 1. Introduction

The United Nations High Commissioner for Refugees (UNHCR) indicates that between 2012 and 2022, the global number of displaced people more than doubled from 42.9 to an estimated 100 million. People fled because of fear of persecution, human rights violations, or violent conflicts such as the Syrian civil war. Germany currently hosts approximately 1.3 million refugees (United Nations Higher Commissioner for Refugees, Global trends Forced displacement, 2021). Of those approximately 890 thousand asylum-seeking individuals who arrived in Germany [1], refugees from Syria formed the main sub-group (Bundesagentur für Arbeit 2016). Recognized refugees in Germany have no legal limitations regarding regional mobility and have unrestricted access to education, the labor market, as well as labor market-related programs supporting integration into the labor market. The legal status is temporary, but in the case of Syrian refugees, the prolongation is performed without difficulties (Bundesministerium für Arbeit und Soziales (BMAS) (2019): Ein Leitfaden zu Arbeitsmarktzugang und -förderung.—FLÜCHTLINGE. Berlin (BMAS). 

Refugees may suffer from multiple stressors before, during, and after migration. The impact of (civil) war such as brute force, bodily harm, and loss of close relatives or others represents potentially traumatic experiences. The additional accumulation of daily hassles, loss of social support, and poor material conditions not only in the receiving country is associated with an increased risk for psychological distress (e.g., symptoms of depression or anxiety) but also for mental disorders such as major depressive disorder or post-traumatic stress disorder (PTSD) [2,3].

The prevalence of PTSD in refugee samples is high. Fazel and colleagues estimated the overall prevalence to be between 8 and 10% in large representative population samples [4]. In a meta-analysis on *n* = 181 surveys, an unadjusted weighted prevalence rate of 30.6% for PTSD and 30.8% for depressive disorders was reported [5]. However, large inter-survey variability depended on sampling methods, sample size, diagnostic period and type of measure, and other factors. 

The major diagnostic criteria of PTSD include a pre-requisite trauma exposition plus intrusions (e.g., intrusive memories pertaining to the trauma), avoidance of associated stimuli (e.g., places, situations), hyperarousal, and others [6,7]. The duration of the disturbance should exceed one month and psychological, social, or occupational dysfunction can be observed.

Similar to depressive disorders, PTSD often results in occupational impairment. PTSD is associated with an average of 42.7 “days out of role” per year, which is comparable to the impairment caused by depressive disorders [8,9]. Furthermore, in samples of natural disaster survivors, current PTSD is not only associated with current unemployment, but also with lower educational attainment and poorer financial status [10]. 

Similar patterns can be observed in refugees exposed to a number of potentially traumatic experiences. In another cross-sectional study on *n* = 586 Cambodian refugees, 20 years after resettlement in the US, the authors also reported a high comorbidity for PTSD and major depression (41%) [11]. In addition, unemployed subjects had a 1.67 and 4.44 times higher odds to suffer from PTSD or major depression, respectively. 

The analysis of the first wave of the Building a New Life in Australia (BNLA) survey on *n* = 2399 refugees from 11 countries found a 1-week screening prevalence of 31% for PTSD (assessed with the 8-item PTSD-8 by Hansen and colleagues) [12]. The four-week screening prevalence for severe mental illnesses (SMI) such as mood and anxiety disorders was 16% (assessed with the 6-item K6 by Kessler and colleagues) [13]. The number of potentially traumatic events and economic stressors pre-migration, including unemployment, showed positive associations with PTSD and SMI. Other post-migration stressors correlating with those disorders were loneliness, discrimination, and family conflicts in Australia [14].

Using nationwide Swedish register data, Di Thiene and colleagues surveyed *n* = 69,515 Swedish-born individuals, refugees, and non-refugee migrants, aged between 19 and 30 years, who had received healthcare for depressive, anxiety, or stress-related disorders in 2009. Compared to the Swedish-born reference group, refugees and migrants had a higher risk for long-term unemployment (>180 days p.a.). The adjusted hazard ratio (aHR) for unemployment were highest for Syrian or Afghan refugees (Ahr = 2.58 and aHR = 2.65, respectively) [15]. A similar large Swedish register study confirmed a higher prevalence of unemployment and mental disorders (except for bipolar disorders) in populations of refugees compared to Swedish-born individuals [16]. 

A more in-depth exploration of a larger dataset (*n* = 4,085,718; aged between 19 and 60 years) from the Swedish register focused on subjects who sought healthcare for specific diagnoses (depressive disorders, bipolar disorder, anxiety disorders, PTSD, other stress-related disorders, and other mental disorders) [17]. The prospective cohort study included the period of time between 2010 and 2013. In general, refugees suffered from mental disorders more often than Swedish-born subjects. PTSD prevalence of 1.3% and 0.1% were found for refugees and Swedish-born subjects, respectively. The comparison between the group of refugees and the Swedish-born subjects without mental disorders (reference-group, RG) revealed an unadjusted hazard risk of 4.67 for long-term unemployment for refugees. After adjustment for socio-economic variables and somatic and mental health at the baseline, the adjusted hazard risk decreased to 1.80. The unadjusted HR for long-term unemployment in Swedish-born subjects with mental disorders, refugees without mental disorders, and refugees with mental disorders were 2.11, 5.57, and 5.97, respectively. After adjustment for socio-economic, work-related, and health factors, the adjusted HR dropped to 1.44, 2.68, and 2.33, respectively. PTSD in Swedish-born subjects or refugees was associated with a 1.41 and 2.25 higher adjusted HR for long-term unemployment, respectively (compared to the RG, unadjusted HR: 2.91 and 6.72, respectively).

Focusing on refugees from the Middle East, longitudinal studies with smaller sample sizes also add to our knowledge on the relationship between mental disorders, PTSD, and unemployment in refugees. Carlson and colleagues studied *n* = 139 refugees from Iran, Iraq, and Lebanon, assessing PTSD, symptoms of anxiety and depression, and wellbeing. During the ten-year follow-up period, PTSD symptoms and emotional distress decreased. PTSD and psychological distress at the follow-up were negatively associated with being (self) employed [18]. A more recent study on *n* = 1924 Syrian refugees resettled in Canada showed a significant association of depressive symptoms with unemployment at the baseline and at year 2. However, when adjusted for other variables, the multinomial logistic regression model did not detect these associations anymore [19]. 

To summarize, research suggests that the unemployment rate is higher for refugees than for subjects originating from the receiving country. Unemployment is a major and interacting risk factor for SMI and PTSD in different refugee populations [20]. In addition, this association, mediating factors, and the prevalence of these mental disorders may vary across time and gender and require further analyses [21].

This study aims at establishing the association between the severity of PTSD symptoms, psychological distress, and labor market integration adjusted for socio-economic factors. The sample consists of Syrian refugees resettled in Germany assessed over the period of five years in order to observe time-varying associations. 

## 2. Materials and Methods

### 2.1. Data

This study combines two sources of data, the “Integrated Employment Biographies (IEB)”, which are register-based data from the German Federal Employment Services (Bundesagentur für Arbeit (BA) [22], and survey-based data from a representative longitudinal sample of young Syrian refugees (IAB WELLCOME Study). As the sample of Syrian refugees was drawn out of the register data, the survey data could be easily matched with register data in case the participants agreed to data merging.

The multi-wave WELLCOME study was designed to explore refugees’ migration experience, their socio-economic background, mental health, and the status of integration into education, training, and work in Germany over time. The following characteristics were applied to the sample to survey young Syrian refugees, who had recently arrived in Germany:

- Arrival in Germany between 2014 and 2016 as civil war refugees;
- Age between 18.0 and 24.9 years, when entering the register data of the Federal Employment Services;
- Clearance of the legal status;
- Entitlement to (temporary) asylum in Germany;
- Enlistment as a job-seeking individual in the unemployment register of the BA in spring 2016 [23].

Enrollment in the unemployment register of the BA provides both institutional support for integration into education or employment in Germany and access to social benefits. A representative sub-sample of *n* = 8604 newly registered refugees was drawn out of 3 consecutive monthly entry cohorts into the German unemployment register [24] (p. 11) aged between 18.0 and 24.9 years when entering the register data. The samples were split into a computer-assisted telephone interview (CATI) and a computer-assisted web interview (CAWI) sub-group, as a telephone number was not available for every member of the sample group in the register data. Individuals for whom the register data provided phone numbers (*n* = 4736/8604) were assigned to the CATI group, and individuals for whom the register data could not provide a phone number were assigned to the CAWI group (*n* = 3868 out of 8604) at wave 1 of the survey. In case the respondents granted access to their phone numbers, the respondents from the CAWI group were assigned to the CATI group in subsequent waves of interviews. 

Sample members were contacted using a standardized procedure. The sampled subjects received a letter of invitation both in German and Arabic language providing detailed information regarding the background of the interview invitation, the aims of the study, and data protection. In the case of the CATI sub-sample, subjects were contacted via telephone within a week after receiving the invitation letter. Respondents could perform the interview in Arabic, English, or German language. Computer-assisted telephone interviews were successfully conducted with *n* = 2328 (= 49.2% of the gross CATI sample *n* = 4736) by trained interviewers of Infas (Institute for Applied Social Sciences, Bonn, Germany). Based on our pre-test experience [24], only Arabic native speakers were accepted to conduct the interviews and the interviews were performed predominantly in Arabic language (98.6%; 1.4% in German language). The average interview duration was 47 min. 

In the case of the CAWI group, the invitation letter included an additional access code to log into the web-based CAWI interview. *n* = 404 of 3868 (10.5%) individuals participated in the web interview. Thus, and in line with the literature, the response rate in general was significantly higher for CATI compared to CAWI [25]. Female and lower qualified respondents participated to a lower degree in the CAWI interviews compared to CATI. However, we found no difference in the prevalence of PTSD screening diagnoses between females (or males) assessed by CATI and females (or males) assessed by CAWI. In addition, no difference was found for PTSD screening diagnoses between lower qualified respondents assessed by CATI and lower qualified respondents assessed by CAWI. In order to take into account any unobserved bias due to the assessment method (CATI/CAWI), we controlled for this factor in the regression models to investigate the association of social factors potentially associated with PTSD. Ex post *n* = 23 (1.1% of *n* = 2080) contacted subjects were excluded from the sample based on the age criterion.

The register data were derived from the Integrated Employment Biographies (IEB), which include episodes of registered unemployment and job search, unemployment or social benefit recipiency, program participation, and all episodes of employment, covered by the German social security system (including marginal employment or internships). 

### 2.2. Questionnaire and Variables

The questionnaire was pre-tested in a pilot study [26]. All variables and measures employed in this analysis were either already validated in English and/or Arabic (e.g., the PTSD-ETI instrument [27]) or were translated according to the EURO-Reeves translation-back-translation protocol (e.g., Hopkins Symptom Checklist-10 or Social Support) [28]. Both the Federal Employment Services’ data officer and the Internal Evaluation Committee of the Institute for Employment Research as well as the Ethics Board of the Medical Faculty of the University Duisburg-Essen approved the study design and the questionnaire. Participation in the interview or completion of the online questionnaire was taken as deemed consent to participate. Panel and matching consent were explicitly requested from the respondents and documented in the data. 

#### 2.2.1. Outcome Variable: Labor Market Integration

As this paper is focused on the process of labor market integration over time, we employ different definitions of employment status as possible outcome variables:

- The first and widest definition includes full-time and part-time employment, as well as marginal employment, apprenticeship training, and being active in an employment program as proxies for labor market integration;
- A second concept allows full-time, part-time, and marginal employment covered by social security as indicators for labor market integration;
- A third definition restricts labor market integration to full-time and part-time employment;
- A fourth definition is restricted to full-time employment.

We assume that full-time employment is the most demanding type of employment, and that full-time employment should respond more severely to mental health, compared to the wider definitions of labor market integration. Table 1 indicates a time-dependent but rather successful process of integration of young Syrian refugees into the German labor market between 2016 and 2021. Following the definitions of labor market integration mentioned above, based on the broadest concept of integration (first definition), 69% of our sample are included in the labor market in 2021. 

Focusing purely on employment contracts, without considering working hours, 64% of our sample are still registered as employed (full-time, part-time, or marginal employed). However, a significant part of the employed are performing marginal jobs (less than 15 h a week). Excluding this latter group, 42% are still employed in full-time or part-time and 31% of the sample are in full-time employment (see Table 1). Table 1 reports individuals’ annual labor market status with respect to the reference data per June 30 of a given year. A reference date typically underestimates individuals’ employment participation over the course of a year (e.g., with respect to temporary periods within a year of observation).

#### 2.2.2. Explanatory Variables

##### Post-Traumatic Stress Disorder—Essen Trauma Inventory (PTSD-ETI)

The PTSD-ETI inventory assesses PTSD according to the Diagnostic and Statistical Manual of Mental Disorders (DSM-IV) criteria A to F. Our results are approved based on excellent scale reliability (Cronbach’s alpha = 0.95), clear factor structure, and good construct validity. The PTSD-ETI validation is based on standardized clinical interviews for PTSD [27] and on different already validated questionnaires assessing PTSD-symptoms and psychopathology (e.g., the Posttraumatic Stress Scale [29]; Peritraumatic Dissociative Experience Questionnaire [30,31]).

Assuming individuals experienced a relatively high level of potential traumatic events in their country of origin, during the flight to Europe, and in the receiving country, we did not employ a trauma list to prevent re-traumatization. At the beginning of the PTSD-ETI section of the questionnaire, the interviewer asked the respondent to recall the worst potentially traumatic event in their biography. The event is assessed based on four questions representing PTSD criterion A. The four event-related questions were divided into two sub-groups (exposure to traumatic event and intense fear/helplessness). The screening diagnosis requires at least one affirmative answer for each of the two sub-groups. These four criterion A questions were presented to all subjects and are a pre-requisite for the PTSD screening diagnosis. Respondents who could not report a potentially traumatic event skipped the following PTSD symptom assessment.

Three out of the original five PTSD-ETI sub-scales were used here: criteria B (intrusions, five items), C (avoidance, seven items), and D (hyperarousal, five items). Criteria E (duration of the disturbance) and F (psychosocial impairment) were omitted. The items of the PTSD-ETI sub-scales were rated on a 4-point Likert scale from 0 (“not at all”) to 3 points (“very often”). According to Tagay and colleagues [27], PTSD is very probable and can be diagnosed, if the A-criterion is met and the total sum of the items reflecting criteria B, C, and D exceeds 26 points. However, due to our population sample and the empirical distribution, we chose a cut-off value of 30 points and above for a positive PTSD screening diagnosis. This cut-off is two standard deviations above the mean of our PTSD-ETI score distribution (top 5%).

The rationale for implementing the PTSD-ETI scale was two-fold. First, the PTSD-ETI was translated into different languages including a transculturally adapted Arabic version, which was used in immigrant or refugee settings [27,32]. Second, the PTSD-ETI is an economic instrument for epidemiological research, as a repeated refusal of answering the PTSD-ETI items led to non-completion of the remaining instrument.

To test the factorial structure of the four items pertaining to criterion A in our sample, these items were subjected to a principal component factor analysis (PCA) using varimax rotation. The data were suitable for PCA (Kaiser–Meyer–Olkin (KMO) = 0.73; significant Bartlett’s test of sphericity; communalities ≥0.550). The PCA revealed a one-factor solution, accounting for 58.6% of the variance. Cronbach’s alpha was 0.76.

Similarly, the 17 items referring to criteria B, C, and D were analyzed by PCA with the number of factors limited to 3 according to the theoretical background (KMO = 0.95; communalities ≥ 0.47). The structure is in accordance with the analysis, which also found an overlap of items for the intrusion and avoidance factors. The 3 factors accounted for 60.4% of the variance, and Cronbach’s alpha was 0.93 for the 17 items.

##### Hopkins Symptoms Checklist 10 (HSCL-10)

The 10-item Hopkins Symptoms Checklist [28] evaluates psychological distress during the previous week. HSCL-10 addresses respondents’ anxiety and depressive symptoms, focusing on the previous two weeks before the interview by applying a 4-point Likert scale. In addition, HSCL-10 is not only a general measure of psychological distress, but it is well established as a screening tool for psychological distress and mental disorders in general in various population samples. A representative Norwegian study differentiated between mentally healthy and disordered subjects, i.e., “psychiatric caseness” or mental disorder, especially anxiety and/or depressive disorder (sensitivity: 89%; specificity: 98%). The Norwegian study reported a Cronbach’s alpha of 0.88, and the study established a cut-off at ≥1.85 points [29]. A Cronbach´s alpha of 0.83 calculated for our refugee sample is in line with findings from the literature and indicates the quality of the Arabian HSCL-10 translation. Due to the literature and the specific population, we follow Strand and colleagues [33] and employ the 1.8 cut-off point as an indicator for a screening diagnosis of a mental disorder.

**Figure 1:**
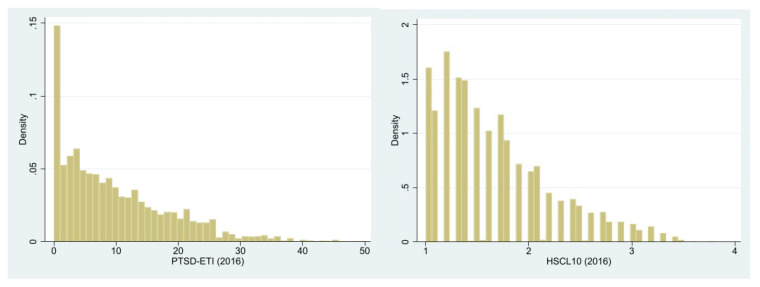
Distribution of PTSD-ETI and HSCL-10 scores in 2016. Source: IAB-WELLCOME study wave 1.

Figure 1 reports the empirical distributions of both measures, PTSD-ETI and HSCL-10 scores in our sample, measured for the year 2016, immediately after our respondents received access to the German labor market.

**Figure 2:**
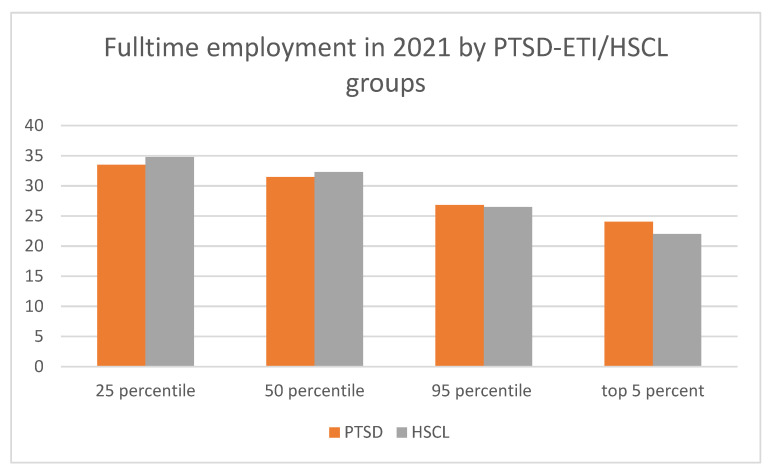
Full-time employment rate by PTSD-ETI and HSCL-10 scores (percentiles) in 2021. Source: IAB-WELLCOME study wave 1; IEB, V16.01.

Figure 2 demonstrates the association of PSDT-ETI and HSCL-10 scores with individuals’ labor market situation. The share of full-time employed corresponds negatively with the severity of PTSD-ETI and HSCL-10 screening scores. 

HSCL-10 and PTSD-ETI scores are correlated; however, the overlap of the PTSD-ETI diagnosis and the HSCL-10 diagnisis is limited (Table 2).

#### 2.2.3. Further Socio-Demographic Variables

The variable age of migration indicates the respondents’ age when leaving the home country. In the case of adolescent refugees, the age of leaving the home country indicates possible involuntary interruptions of individuals’ educational pathway. Staying in a third country whilst migrating might indicate both adverse situations of quality of life regarding socio-economic security and health-related dimensions. 

The year of arrival in Germany indicates both the readiness of Germany to integrate refugees and the competition within the refugee groups. In 2014, a proportionally small group of refugees arrived in Germany and Germany was less prepared to host and integrate large numbers of refugees. A majority of refugees arrived in 2015. Refugees, who arrived in 2016, report either longer migration time or report that they had left their home country when the first groups had already arrived in Germany. In addition, Germany was better prepared to host and integrate the new arriving refugees.

Migration-related debts to friends, relatives, or formal finance providers might burden individuals’ process of integration. Degrees from general and vocational education and training before arriving in Germany indicate refugees’ preparedness for the German labor market. As Table 3 demonstrates, the average level of general education in our sample of Syrian refugees is rather high (70% with degrees higher than lower secondary education); however, the extent of vocational and academic training is low.

Despite the high level of general and vocational education already attained before arriving in Germany, the intention to pursue a degree in Germany is rather high. A total of 68% preferred academic study to working or vocational training.

In line with the general level of education, refugees report a good level of mathematical skills. However, refugees only report a moderate level (31% good to excellent) of German language proficiency in 2016, which is not particularly surprising due to the European at-random allocation of refugees to countries and regions. The longitudinal data, however, indicate that the refugees improved their language proficiency well and fast within the subsequent years.

Respondents’ socio-economic status, indicated here by the fathers’ educational level, appears to be rather high, as 32% of the respondents report paternal academic qualification.

Characteristically, the majority of Syrian refugees arriving in that period of time were male (86%) and of Muslim confession (92%).

The refugees were distributed more or less evenly over the German regions. The federal states of Germany reflect the overall population distribution. Migration of refugees over time is reduced (with respect to place of living, however, with slightly shares of regional mobility within Germany with respect to place of work).

To capture possible survey effects, we controlled for mode variation and distinguished CAWI interviews, CATI interviews with males, or CATI interviews with female interviewers, respectively (see Table 3).

### 2.3. Modeling

Ordinary least square regression models (OLS) were estimated to include all explanatory and control variables into the models simultaneously. Additionally, we controlled for the factor of residential area. Average marginal effects and marginal effect plots were calculated. All estimates were performed with Stata 17. 

## 3. Results

**Figure 3:**
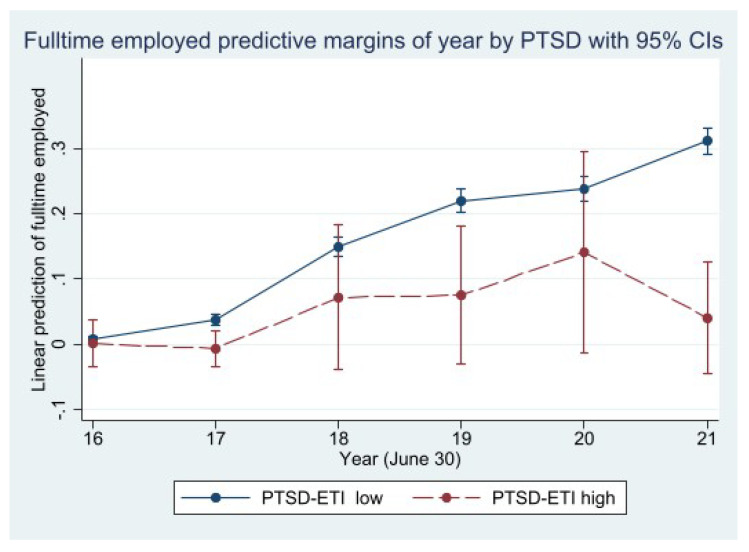
Linear prediction of full-time employment by PTSD-ETI screening diagnosis—2016-2021. Source: IAB-WELLCOME study wave 1; IEB, V16.01.

Figure 3 displays the average marginal effects of a PSTD screening diagnosis on individuals’ employment probability. The marginal effects are calculated under control of a rich set of covariates (see Table A1 in Appendix A). The model applied differentiates between two groups of interest: (a) individuals scoring above the PTSD cut-off value of 30 (PTSD screening diagnosis) and (b) individuals with a score equal or below the cut-off value. The high-PTSD group shows significantly lower employment probabilities over the observation period 2016–2021. Especially at the beginning of this period, but also over the course of time, the high-PTSD group shows significantly lower employment probabilities compared to the low-PTSD group. This gap is expanding at the end of the observation period, during which the overall employment participation was increasing. In contrast to the high-PTSD group, the low-PTSD group shows a steady increase of employment probabilities (see Table A1 in Appendix A for full models). 

**Figure 4:**
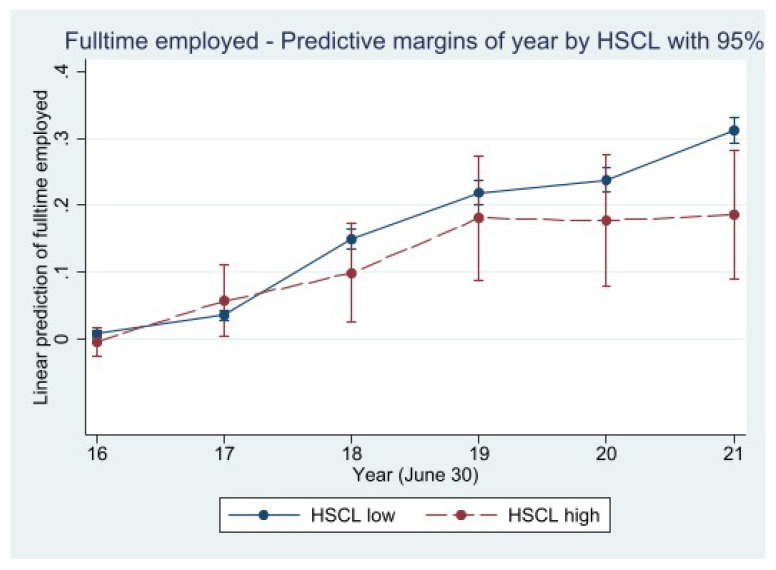
Linear prediction of full-time employment by mental disorder screening diagnosis—2016–2021. Source: IAB-WELLCOME study wave 1; IEB, V16.01.

The full model predicting full-time employment by a mental disorder screening diagnosis (HSCL-10) and under control of independent variables indicates a similar but less pronounced effect on labor marktet integration (Figure 4). For subjects with a high probability for a mental disorder diagnosis, as defined by the respective HSCL-10 cut-off value of ≥1.85 points, the register data report lower full-time employment compared to individuals with lower initial HSCL-10 values, as reported in 2016. Similar to the analysis pertaining to the PTSD-ETI, we observe a significant effect of lower full-time employment in 2021 for subjects with a high probability of suffering from a mental disorder. Again, the predictive margins are based on the full model including the whole set of control variables (see Table A2 in Appendix A).

**Figure A1:**
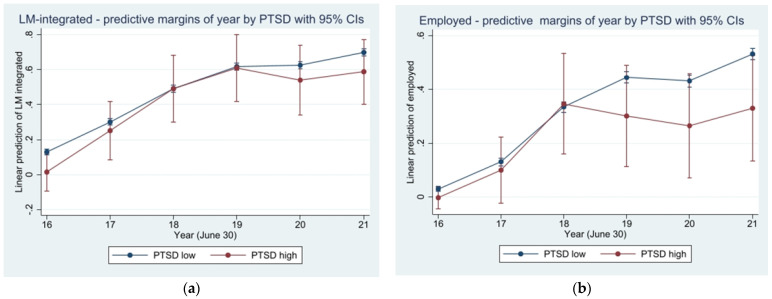

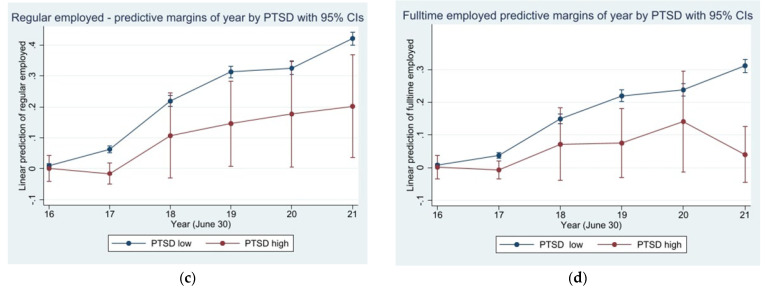
Predictive margins of year by PTSD screening diagnosis (ETI) on labor market integration. (**a**) Labor market integrated (wide definition). (**b**) Employed (full-time, part-time, or marginal employed). (**c**) Regular (full-time or part-time) employed. (**d**) Full-time employed. Source: IAB-WELLCOME study wave 1; IEB, V16.01.

**Figure A2:**
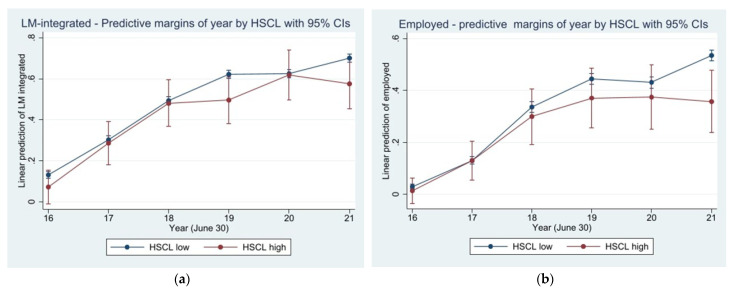

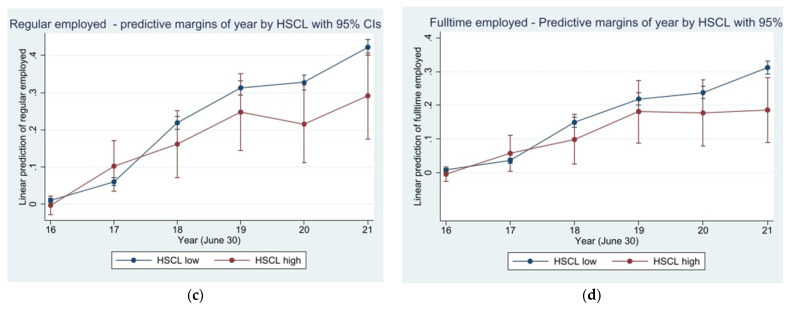
Predictive margins of year by mental disorder screening diagnosis (HSCL). (**a**) Labor market integrated (wide definition). (**b**) Employed (full-time, part-time, or marginal employed). (**c**) Regular (full-time or part-time) employed. (**d**) Full-time employed. Source: IAB-WELLCOME study wave 1; IEB, V16.01.

Regarding the individuals´ employment status, we observed for both models that individuals’ migration-related characteristics lose their initial discriminating power over time. We found two exemptions: The chances of individuals to be in full-time employment are higher for those who were older than the average age found in the sample at the beginning of their migration process. We assume these individuals had already finished their educational track in the home country and might have already started their employment career. The second and related exemption pertains to educational aspirations. Respondents, who reported a preference for future attendance at a university instead of job search or vocational training, showed a significantly lower employment probability. In our sample, educational aspiration is correlated with educational degrees already attained in the home country and with high levels of mathematical competences. That indicates that these indiviuals are prepared for academic studies and are willing to maintain their aspiration (see Table A1 and Table A2 in Appendix A). Figure A1 and Figure A2 in Appendix A additionally demonstrate the association between type of labor market integration over time and the PTSD-ETI and HSCL-10 diagnosis.

## 4. Discussion

There is an extensive bibliography on the consequences of unemployment on mental health [34]. However, studies on the effects of mental health on finding a job are less frequent [35,36]. Migration movements due to economic or political reasons or fleeing from war and violence and the transition into the labor market in receiving countries offer an appropriate framework for this type of study. The present article focuses on the analysis of the association between mental health and participation in the labor market. Assessing a sample of young Syrian refugees, who have arrived in Germany between 2014 and 2015, we found an association between the probability of the refugees finding a full-time job and the aggravation of mental health issues deriving from the migration process, such as post-traumatic stress disorder and severe psychological distress as seen in mental disorders. These young refugees have experienced violence in the country of origin, harsh conditions during the emigration to the receiving country, and difficulties in adapting to a new social and cultural environment. Some studies estimate that around 20% of migrants suffer from anxiety, depression, or PTSD [37,38,39]. In our sample, we calculated a 7% PTSD-ETI screening diagnosis and a 29% screening diagnosis for mental disorders with respect to HSCL-10. Although studies carried out with small samples of refugees have obtained higher prevalence rates, the prevalence rates obtained for this sample are similar to those observed in studies carried out with large and representative samples [4,26]. It is to be expected that the refugees’ experiences will have consequences for their integration into the labor market of the receiving country.

The degree of integration of these young refugees into the labor market increased throughout the observed period (2016–2021), reaching an employment rate of 69% in 2021, of which the majority (30% of the total sample) held a full-time job. With respect to western societies, Brell and colleagues reported consistently lower employment rates over time for refugees, compared to labor migrants [40]. In Germany, active labor market participation can be a pre-condition for the residence permit of labor migrants. In contrast, the residence status of a refugee depends on the individual´s need for protection, which may be connected either to access to education or to the labor market. The integration into education of the labor market is not a pre-condition for a (temporary) residence permit. Thus, in Germany, employment rates of refugees typically are lower compared to those of labor migrants. This specific legal situation for refugees in Germany might also contribute to the results reported by Brell and colleagues, i.e., the labor market integration in Germany is in the lower third of the integration distribution in Western industrialized countries [40]. Kosyakova and Kogan [41] reported similar results. Considering these findings from the literature, the sample of young Syrians in our study seems to be integrated into the labor market quite well.

Nevertheless, integration is affected by traumatic experiences [15]. Di Thiene and colleagues reported significant differences within the labor market for different refugee groups and natives with respect to the mental health distribution. These findings are in line with results from this study, where we could show that refugees with higher levels of PTSD experience more difficulties in finding full-time employment throughout the entire period analyzed between 2016 and 2021. In contrast, those refugees with lower levels of PTSD symptoms show a higher continued probability of finding full-time employment throughout the period analyzed. 

Regarding those subjects with a screening diagnosis of a mental disorder, a similar but less pronounced association was found. The likelihood of having a full-time job in 2021 was significantly lower for those with “psychiatric caseness” or high levels of depression and anxiety. These data are consistent with the evidence provided by other studies that indicate the association between aggravation of mental health and a more fragile position in the labor market [36,42,43]. On the other hand, our findings add to those provided by other studies, in which the aggravation of mental health issues associated with the refugees’ experiences is the explanatory factor for worse labor integration when compared to other categories of migrants [44].

The only significant predictors of employment probabilities regarding individual characteristics were the age at which the country of origin was left and the educational aspirations. The probability of having a full-time job in 2021 was lower for those who showed a preference for academic training, compared to those who were oriented towards job search or vocational training. In this sense, the high educational level of this sample is remarkable and coincides with findings from other studies, in which the high level of qualification of refugees, who arrive in the European Union countries, is highlighted [45].

## 5. Strengths and Limitations

This paper benefits from high-quality survey data as well as from longitudinal register data obtained from young Syrian refugees. The register data include rich and precise information on the individual labor market status and income. The longitudinal register data are available up to the year 2021: as high matching consent (97%) was obtained, the register data could be employed for almost all participants. The data assessed for this study are of very high quality given the adequate psychometric properties of the mental health measures used, the high response rates, and the absence of biases in the final sample in comparison to the register-based gross sample. 

As a limitation, we have to consider that we limited our sample due to language restrictions of young Syrian refugees getting access to the labor market in Germany in spring 2016. This group is representative of the majority of young Syrian immigrants who have arrived between 2014 and 2016, which is the peak of migration from the Middle East to Germany. However, it does not include arrivals as of the middle of 2016 and onwards. Unfortunately, the information regarding refugees is school based. Educational data including university degrees are insufficiently reported in the register data.

## 6. Conclusions

Getting a job is a key factor not only for obtaining economic resources, but also for achieving social inclusion. The integration of refugees into the labor market not only implies an individual benefit, but also has positive consequences for the social and economic development of the receiving country itself.

The results of this study indicate that the deterioration of mental health associated with the experiences during the migration process is one of the main obstacles to the full labor integration of refugees. This fact leads us to point out the importance of developing public employment policies aimed at this social group which could contribute to reducing the negative consequences of the migration process on their mental health.

## Figures and Tables

**Table 1:** Respondents’ employment status 2016–2021.

Definition of Employment Status	2016	2017	2018	2019	2020	2021
Any form of employment (incl. marg. employed, trainees, and scheme-based employment contracts)	12.7%	29.7%	49.3%	61.4%	62%	69.3%
Employed (incl marg. employed)	3%	14%	38%	52%	54.7%	63.5%
Employed (full- and part-time employed)	0.5%	6.1%	21.9%	30.9%	32.8%	42.1%
Full-time employed	0.3%	3.5%	14.9%	21.8%	24.2%	31.2%
Total (*n* = 100%)	1794	2415	2335	2280	2109	2190

Source: IAB-WELLCOME study wave 1; IEB V16.01. WELLCOME- population; annual labor market status at reference day: June 30.

**Table 2:** Association of HSCL-10 and PTSD-ETI screening diagnoses in 2016 (%).

	PTSD-ETI Screening Diagnosis		
HSCL-10	No PTSD- Diagnosis (Below 30 Points)	PTSD Screening Diagnosis (>= 30 Points	Total
No diagnosis of mental disorder (Below 1.8 points)	68.04	2.69	70.73
Screening diagnosis of a mental disorder (1.8 points and above)	25.07	4.21	29.27
Total	93.11	6.89	100

Source: IAB-WELLCOME study wave 1. Pearson chi2 (1) = 80.6020; Pr = 0.000.

**Table 3:** Explanatory variables—descriptive.

Explanatory Variables	Obs.	Mean	Std. Dev.	Min	Max
**Key variables**					
PTSD screening diagnosis (ETI)	2190	1.63	0.61	1.00	4.00
Mental disorder screening diagnosis (HSCL-10)	2190	1.66	0.54	1.00	3.80
**Further socio-demographic variables**					
Male	2190	0.87	0.34	0.00	1.00
Muslim	2190	0.92	0.27	0.00	1.00
Age at immigration into Germany	2190	20.15	2.07	12.42	24.42
Long-term stopover in third country (1 = yes)	2190	0.35	0.48	0.00	1.00
Year of immigration					
2014	2190	0.04	0.20	0.00	1.00
2015	2190	0.81	0.39	0.00	1.00
2016	2190	0.15	0.36	0.00	1.00
Migration related depts	2190	0.21	0.41	0.00	1.00
General education					
No degree	2190	0.06	0.23	0.00	1.00
Primary degree	2190	0.08	0.28	0.00	1.00
Lower sec degree	2190	0.29	0.45	0.00	1.00
Upper sec degree	2190	0.46	0.50	0.00	1.00
Post sec voc. degree	2190	0.06	0.24	0.00	1.00
Academic degree	2190	0.05	0.21	0.00	1.00
Study intention at arrival	2190	0.68	1.53	0.00	9.00
Math-score (very good)	2190	0.27	0.45	0.00	1.00
German language proficiency					
Excellent	2190	0.01	0.11	0.00	1.00
Very good	2190	0.07	0.26	0.00	1.00
Good	2190	0.23	0.42	0.00	1.00
Sufficient	2190	0.43	0.50	0.00	1.00
Not sufficient	2190	0.25	0.44	0.00	1.00
Father academic educated					
No	2190	0.76	0.43	0.00	1.00
Yes	2190	0.11	0.32	0.00	1.00
Missing info	2190	0.13	0.33	0.00	1.00
Mode of interview					
CAWI	2190	0.13	0.34	0.00	1.00
CATI male interviewer	2190	0.52	0.50	0.00	1.00
CATI female interviewer	2190	0.35	0.48	0.00	1.00
Annual reference date (June 30)					
2016	13,123	0.14	0.34	0.00	1.00
2017	13,123	0.18	0.39	0.00	1.00
2018	13,123	0.18	0.38	0.00	1.00
2019	13,123	0.17	0.38	0.00	1.00
2020	13,123	0.16	0.37	0.00	1.00
2021	13,123	0.17	0.37	0.00	1.00

Source: IAB-WELLCOME study wave 1; IEB, V16.01.

## Data Availability

The entire dataset cannot be made publicly available due to data protection legislation (covered by Social Code Book X §80).

## Appendix A

**Table A1:** Effects of PTSD screening diagnosis in 2016 on being employed—2016–2021—OLS regression.

	2016	2017	2018	2019	2020	2021
**Male** (1 = yes)	0.0023	0.0328 **	0.1244 ***	0.1909 ***	0.1893 ***	0.2733 ***
**Muslim** (1 = yes)	0.0046	−0.0026	0.0318	0.0173	−0.0207	−0.0264
**Migration age**	0.0014 +	0.0057 *	0.0116 **	0.0107 *	0.0127 *	0.0136 *
**Living in third country** (1 = yes)	0.0074 *	0.0120	0.0117	0.0204	0.0225	0.0304
**Arrival year** (2014 = ref)						
2015	−0.0193 **	−0.0447 *	−0.0036	−0.0316	−0.0237	0.0092
2016	−0.0211 **	−0.0579 *	−0.0164	−0.0310	−0.0194	−0.0111
**Migration debts** (1 = yes)	−0.0006	0.0071	0.0046	0.0003	0.0013	0.0047
**Edu degree** (ref = acad degree)						
No degree	0.0190 +	−0.0142	0.0132	−0.0515	−0.0113	−0.0936
Primary degree	0.0003	0.0091	−0.0550	−0.0155	−0.0291	−0.1276 *
Lower sec degree	0.0031	0.0053	−0.0271	−0.0339	−0.0453	−0.1214 *
Upper sec degree	0.0053	0.0052	−0.0405	−0.0506	−0.0832 +	−0.1280 **
Post sec voc	−0.0001	0.0080	−0.0232	−0.0982 +	−0.0869	−0.1920 **
**Study Aspiration** (1 = yes)	−0.0048	−0.0156 +	−0.0471 **	−0.0942 ***	−0.0910 ***	−0.1083 ***
**Math skills very good/excellent**	0.0054	0.0083	0.0149	0.0014	−0.0044	−0.0071
**German language** (1 = excellent/5 no)	−0.0036 *	−0.0104 *	−0.0154 +	−0.0092	−0.0147	−0.0069
**Father academic edu** (1 = yes)	−0.0069	−0.0210	0.0083	−0.0014	−0.0143	−0.0281
cati = 1	−0.0107 *	0.0132	−0.0203	0.0166	0.0195	0.0033
**PTSD** (cat)	−0.0023	−0.0052	−0.0214	−0.0510 **	−0.0384 +	−0.0643 **
Cons	0.0094	−0.0243	−0.0618	0.0397	0.0633	0.1102
Number of observations	1607	2151	2078	2027	1872	1942
Degree of freedom	20.000	20.000	20.000	20.000	20.000	20.000
R2 adj	0.009	0.012	0.030	0.053	0.042	0.082

Data Source: IAB WELLCOME wave 1+ IEB V16.01. Note: significance levels: + *p* < 0.1,* *p* < 0.05, ** *p* < 0.01, *** *p* < 0.001.

**Table A2:** Effects of mental disorder screening diagnosis (HSCL, anxiety, and depression) in 2016 on being full-time employed—2016–2021—OLS regression.

	2016	2017	2018	2019	2020	2021
**Male** (1 = yes)	0.0021	0.0322 **	0.1224 ***	0.1857 ***	0.1914 ***	0.2720 ***
**Muslim** (1 = yes)	0.0047	−0.0021	0.0201	0.0163	−0.0229	−0.0300
**Migration age**	0.0014 +	0.0057 **	0.0121 **	0.0122 *	0.0144 **	0.0142 *
**Living in third country** (1 = yes)	0.0072 *	0.0119	0.0169	0.0230	0.0231	0.0390 +
**Arrival year** (2014 = ref)						
2015	0.0185 **	−0.0423 *	−0.0011	−0.0220	−0.0257	0.0014
2016	−0.0201 *	−0.0554 *	−0.0166	−0.0269	−0.0205	−0.0200
**Migration debts** (1 = yes)	−0.0007	0.0071	0.0082	0.0035	0.0072	0.0114
**Edu degree** (ref = acad degree)						
No degree	0.0178 +	−0.0137	0.0183	−0.0446	−0.0077	−0.1015
Primary degree	0.0006	0.0100	−0.0531	−0.0046	−0.0212	−0.1187 +
Lower sec degree	0.0031	0.0051	−0.0268	−0.0257	−0.0361	−0.1199 *
Upper sec degree	0.0053	0.0054	−0.0382	−0.0437	−0.0753	−0.1204 *
Post sec voc	0.0003	0.0074	−0.0243	−0.0979 +	−0.0860	−0.1930 **
**Study Aspiration** (1 = yes)	−0.0046	−0.0151 +	−0.0451 **	−0.0915 ***	−0.0866 ***	−0.1067 ***
**Math skills very good/excellent**	0.0052	0.0082	0.0150	−0.0003	−0.0065	−0.0074
**German language** (1 = excellent/5 no)	−0.0036 *	−0.0103 *	−0.0143	−0.0097	−0.0159	−0.0064
**Father academic edu** (1 = yes)	−0.0069	−0.0205	0.0140	−0.0024	−0.0158	−0.0224
**HSCL** (cat)	−0.0001	−0.0004	−0.0249 +	−0.0357 *	−0.0157	−0.0630 ***
Cons	0.0047	−0.0360	−0.0649	−0.0249	−0.0107	0.0964
Number of observations	1637	2191	2120	2067	1911	1980
Degree of freedom	20.000	20.000	20.000	20.000	20.000	20.000
R2 adj	0.008	0.011	0.032	0.052	0.042	0.083

Data Source: IAB WELLCOME wave 1+ IEB V16.01. Note: significance levels: + *p* < 0.1, * *p* < 0.05, ** *p* < 0.01, *** *p* < 0.001.
